# ‘Doc, can I fly to Australia?’ A case report and review of delirium following long-haul flight

**DOI:** 10.1192/pb.bp.115.052209

**Published:** 2017-02

**Authors:** Thomas McCabe

**Affiliations:** 1 NHS Lanarkshire, Scotland, UK

## Abstract

Air travel is now a common feature of most of our elderly population's lives. There is little by way of warnings, rules or recommendations for our patients with psychiatric diagnoses, in particular dementia, who intend to travel by plane, in contrast to other specialties. In this article I highlight an adverse outcome of long-haul air travel as a result of delirium and resulting accelerated decline in overall cognitive function. I review literature related to the topic and suggest ways to minimise precipitating factors for stressors prior to and during flights. This article suggests that more thought should be given to the title question.

Short-haul and long-haul air travel are now commonplace among the elderly population. Evidence suggests such travel is on the increase from the late 1990s, with as many as 1 in 5 people above the age of 65 passing through the main UK international airports in 2014.^1,2^ Little thought is given to short weekend breaks abroad or long-distance holidays with family with mental and physical impairments. In-flight adverse events are difficult to ascertain from information and figures produced by airlines, and apart from the well-recognised morbidity they are not well reported.^3^ Syncope, respiratory problems and vomiting continue to provide the bulk of in-flight emergencies, with around 1 medical emergency occurring in every 600 flights.^4^ There are no data focusing on the elderly population or people with a diagnosis of mental illness, although one recent source cited ‘anxiety’ as a reason for non-traumatic in-flight complaints.^5^

In this case report I highlight an incident where a delirium has had a significant impact on an elderly patient, resulting in an accelerated decrease in function, long-term impairment and associated symptomatic control with medications that otherwise could have been avoided. I will highlight ways of minimising chances of negative outcomes following air travel in the elderly population.

## Case presentation

A 73-year-old man was initially referred to the memory clinic in December 2013 with a history of gradual deterioration of short-term memory and increased dependence on family over an approximate 18-month period. This was with a background of stable multiple sclerosis with no other medical history of note. He was functioning well owing to a supportive family and positive routine activities undertaken mostly with his wife and had a strong academic background which could have contributed to masking of cognitive defects. He was given a diagnosis of a mild cognitive impairment based on impaired short-term memory, as evidenced by clinical evaluation and formal cognitive testing. He scored 80/100 on the Addenbrooke's Cognitive Examination III; most points were lost in the memory parts (where he scored 10/26) and less so in the fluency part (scoring 10/14) of the test. He was referred to neuropsychology, for brain imaging and was to be seen back in the clinic in 6 months' time. There were no treatable cardiovascular risk factors at this point; however, the patient was counselled on exercise and diet as well as basic activities to promote cognitive training in keeping with present guidance. The patient and his wife were informed it was difficult to give prognosis and although evidence is varied, a person with amnesic (memory loss) mild cognitive impairment would be at around 1 in 5 risk of being diagnosed with dementia at a later stage. From this presentation and clinical evaluation, it was thought that an Alzheimer's dementia was the most likely future diagnosis.

The patient then travelled by plane from Scotland to Australia, which involved a short connecting flight to London. On descent from the air to Australia, he experienced an episode of agitation and bizarre, nihilistic delusions about Nazis taking over the plane. After landing, he was admitted to a large, well-known teaching hospital in Australia. He continued to exhibit challenging behaviours such as agitation, wandering and resistance to attempts at basic care from nursing staff. The patient required assistance with feeding and one-to-one nursing care for the majority of the in-patient stay.

The patient's medical investigations included a positron emission tomography (PET) scan, magnetic resonance imaging (MRI) brain scan, lumbar puncture, electrocardiogram (ECG), chest X-ray, urinalysis, basic blood tests and whole-body computed tomography (CT) over the course of admission without an obvious cause being found for such a dramatic decline and previously unseen behaviours. Of note, any cerebrovascular accident, pneumocephalus, acute kidney injury, external and middle-ear disease and sepsis were ruled out on admission. It was noted that there were no particular risk factors in terms of family history, smoking, hypertension, dyslipidaemia and diabetes which could have increased the risk of most of the proposed diagnoses. Basic observations, including oxygen saturations, were largely unremarkable throughout his admission. He was reviewed by both the neurology and neuropsychiatric teams who came to a joint conclusion that he was experiencing a delirium and previously undiagnosed Alzheimer's dementia.

He was treated with antipsychotics and given a short trial of intravenous steroid given the history of multiple sclerosis (although not indicated by way of imaging) without any improvement in clinical state.

The patient stabilised enough for repatriation to the UK with a nurse escort after approximately 4 months of in-patient care. At this stage he showed significant deterioration in executive functioning from baseline assessment, limited capacity for new learning, confabulation and required assistance with all personal needs. On descent of the aircraft the patient again deteriorated, with disorientation, agitation and paranoid features once again predominant. Admission to hospital and further investigations gave us no obvious clues to an ongoing acute event. Brain imaging in both Australia and the UK remained unchanged with CT showing generalised atrophy and MRI revealing a minor degree of small vessel disease and smaller than expected hippocampal volume, and suggested Alzheimer's as the only radiological explanation for the presentation.

### Outcome

The episode has had a significant effect on the patient's level of functioning and a sizable knock-on effect on his family. He is now a patient in a National Health Service (NHS) long-term care facility requiring assistance with all basic activities of daily living and without much by way of coherent or meaningful conversation. He continues to be managed with antipsychotic medications and benzodiazepines which allow him to be settled on the ward and nursing staff to assist with his needs. It is difficult to ascertain whether there has been any further deterioration in the patient's disease process, however, there has not been any improvement seen. His family continue to harbour feelings of guilt at the original decision to fly to Australia without consultation with medical staff and disappointment at being unable to care for the patient in their home.

My overall aim for writing this case is for the reader to acknowledge the case of a patient with a likely dementia who has had a stark and accelerated decline in function as a result of a change in environment and residual delirium after going on two long-haul flights. Pinpointing the precise mechanism for the delirium continues to be a challenge owing to the number of medical staff involved and the difficulties with communication between continents. Prolonged hypoxia or changes in cabin pressure would seem to be the most obvious causative factors for the delirium given the collateral history, with particular focus on symptoms becoming pronounced on descent. However, this is without any firm scientific basis and is not backed up by anything discovered on clinical examination or investigation.

## Discussion

Air travel has become a normal part of everyday life in the UK, with Heathrow, the third-busiest airport in the world, estimated to see approximately 1300 take offs and landings in an average day.^6^ The advent of ‘no frills’ airlines in the mid 1990s has seen a marked rise in short-haul flights and regional airports have expanded as a result. Combined with the total standardised prevalence of dementia syndrome in the 65+ population, which is thought to be 7.1% at most recent estimates,^7^ air travel is now a common component for a sizable amount of the older population.

From a literature search it would appear that air travel in the elderly population is a safe practice, given that there is little evidence to suggest otherwise. However, there have been a number of reports recently of people with dementia getting lost in airports resulting in national press- and social media-aided searches.^8,9^ Roberto Castiglioni, an adviser to the UK Civil Aviation Authority, has described the impact dementia could have on air travel as ‘a ticking time bomb that medical research and the travel industry are yet to address’.^10^

There is one reported case of an older man in Australia, a seasoned traveller, who in 2009 developed delirium on a long-haul flight and spent a long period of time as an in-patient as a result.^11^ The authors state that the patient's decline was precipitated by air travel, but they do not expand on this. They propose that a brief cognitive screening tool to be used prior to travel be developed.

A useful review, also from Australia,^12^ summarises the physical hazards associated with air travel and states that people with early dementia may be more prone to developing delirium in flight. It sets out practical ways to minimise this risk.

In contrast to the above there are strict criteria for air travel for physical ailments which are well publicised and adhered to by all the major airlines. For example, the Civil Aviation Authority suggest 14 days have passed prior to air travel following a coronary artery bypass grafting (CABG) procedure and most airlines will not allow women with single pregnancies beyond 36 weeks to travel with their companies. Compare this with the less stringent statement that they would have ‘concern’ (rather than instruction not to travel) with patients who may exhibit or develop ‘disorganised and disruptive behaviours’ in flight, as set out in the Civil Aviation Authority's ‘fitness to fly’ guidance.^13^ This is a likely reflection on the lack of morbidity and mortality figures available on the topic combined with the unpredictable course and variable stages of dementia and degree of severity of delirium.

It should also be noted that not only this case described but the others mentioned in the discussion involved movement to and from Australia. It would seem unlikely that this alone is a causative factor and indeed it is the length of flight or descent from high altitude that increases the likelihood of deterioration, but it is worth bearing in mind if the overall topic expands, as some have predicted.

## Recommendations

Table 1 sets out practical ways to help minimise any air travel-related situations which may potentially cause upset to a patient. These can be addressed prior to travel by input from general practitioners (GPs) and optimising control of pre-existing conditions as well as assistance with travel insurance practicalities. Contact with airports and airlines with the aim of reducing transit time through airports and assistance getting on the plane as well as sensible seating choice (i.e. more leg room, access to lavatory) may also aid in reducing potential stressors.

**Table 1:** A summary of recommendations

Pre-flight	In-flight
Attend GP	Stay well hydrated

Optimise chronic conditions	Comfortable clothing

Travel insurance	Familiar distractions

Consider group tours	Inform cabin crew

Assistance in airport	Assistance on/off flight

Extra leg room request	Avoidance of alcohol/unfamiliar foods

Minimise time through security checks	Aisle seating request

GP, general practitioner.

I believe GPs and old age psychiatrists should consider more extensive counselling when the title question is asked and indeed any questions around travel could be pre-empted by medical staff. Patients and carers should be made aware of the dangers posed by hypoxia, changes in pressure environments and barotrauma and the potential for adverse outcomes that these can have on mental state, particularly in those with pre-existing respiratory and ear, nose and throat (ENT) conditions. Little research has been carried out into the overall use, benefit or otherwise of anxiolytics in elderly (or indeed anxious) flyers and this should be explored. Informal discussions with colleagues in the community would suggest the use of benzodiazepines is accepted and relatively common.
